# Trends in Clinical Cardiac Photon-Counting Detector CT Research: A Comprehensive Bibliometric Analysis

**DOI:** 10.3390/diagnostics15040504

**Published:** 2025-02-19

**Authors:** Arosh S. Perera Molligoda Arachchige, Federica Catapano, Costanza Lisi, Jad El Choueiri, Francesca Pellicanò, Stefano Figliozzi, Letterio S. Politi, Marco Francone

**Affiliations:** 1 Department of Biomedical Sciences, Humanitas University, Via Rita Levi Montalcini 4, Pieve Emanuele, 20090 Milan, Italy; 2 IRCCS Humanitas Research Hospital, Via Manzoni 56, Rozzano, 20089 Milan, Italy

**Keywords:** photon-counting detector CT, bibliometric, meta-analysis, clinical, applications, cardiac imaging

## Abstract

Photon-counting detector computed tomography (PCD-CT) represents a significant advancement in radiological imaging, offering substantial potential for cardiac applications that remain partially underexplored. This bibliometric analysis investigates the evolution and current clinical application of cardiac PCD-CT by examining research trends from 2019 to 2024. The analysis aims to understand the development of this technology, its clinical implications, and future directions. A comprehensive literature search was conducted using databases such as PubMed, EMBASE, Scopus, and Google Scholar, yielding 984 records. After removing duplicates and applying inclusion criteria, 81 studies were included in the final analysis. These studies primarily focused on coronary artery calcium scoring, coronary atherosclerotic plaque assessment, and coronary artery stenosis quantification. The findings indicate a significant upward trend in the number of publications, peaking in 2023. The bibliometric analysis revealed that the USA, Germany, and Switzerland are the leading contributors to PCD-CT research, with prominent institutions like the Mayo Clinic and the University of Zurich driving advancements in the field. The NAEOTOM Alpha by Siemens Healthineers, being the only commercially available PCD-CT model, highlights its central role in cardiac imaging studies. Funding for PCD-CT research came from various sources, including industry leaders like Siemens and Bayer, as well as governmental and academic institutions. The analysis also identified several challenges that PCD-CT research faces, including the need for larger patient cohorts and broader geographical representation. In conclusion, the rapid growth of cardiac PCD-CT research underscores its transformative potential in clinical practice. Continued investment, collaboration, and extensive research are essential to fully harness the benefits of PCD-CT.

## 1. Introduction

Photon-counting detector computed tomography (PCD-CT) marks a major advancement in radiological imaging, delivering substantial improvements compared to traditional CT techniques. Photon-counting detectors are composed of specialized semiconductor materials, such as cadmium telluride (CdTe) or cadmium zinc telluride (CZT), which have excellent X-ray absorption properties. These materials allow for the efficient conversion of X-ray photons into electrical signals while reducing noise, allowing for high spatial resolution and spectral imaging capabilities. This results in superior image quality, enhanced tissue differentiation, and the ability to perform multi-energy imaging, all achieved with lower radiation doses compared to conventional CT detectors [1,2,3]. These technological advances hold significant potential to overcome several inherent limitations of conventional CT, particularly in the cardiovascular field. Cardiac imaging is particularly challenging due to the heart’s complex, constantly moving structures and the small size of the coronary arteries. Traditional CT modalities, despite their advancements, often struggle with issues such as limited spatial resolution, excessive image noise, and artifacts, particularly in patients with high coronary calcification or those requiring ultra-low radiation doses [2,4]. PCD-CT emerges as a potent solution to these issues, with its ability to reduce electronic noise, enhance iodine contrast-to-noise ratio (CNR), and mitigate beam-hardening and metal artifacts, thus improving diagnostic accuracy and patient outcomes [1,2,4]. Furthermore, the inherent spectral information provided by photon-counting detectors enables advanced tissue characterization [5]. This is because myocardial diseases involve changes in tissue composition, such as fibrosis, edema, or infiltration of fat, iron, or amyloid. Fibrosis is common and can be interstitial (diffuse) or replacement fibrosis (scar tissue). Interstitial fibrosis is measured using myocardial extracellular volume (ECV), with cardiac magnetic resonance (CMR) as the gold standard, but recent studies show CT can be a valid alternative [6,7]. Both single-energy and dual-energy CT methods effectively quantify ECV, with dual-energy PCD-CT offering lower radiation doses and strong correlation with CMR [6,7]. While CT’s ability to detect replacement fibrosis (scarring) remains limited, PCD-CT has shown promise, demonstrating accurate scar detection in initial studies involving canine models [8]. Similarly, it also improves the differentiation of the atherosclerotic plaque component, being able to precisely characterize high-risk lipid-rich plaques prone to rupture, which allows for earlier and more precise intervention [9,10]. The clinical potential of PCD-CT has been under investigation for over a decade, with early experimental systems demonstrating promising results. However, the technology faced significant hurdles related to detector performance under high X-ray flux rates [1,2,4]. It was not until 29 September 2021 that the Food and Drug Administration (FDA) cleared the first PCD-CT developed by Siemens Healthineers for clinical use, offering real-world validation of its potential benefits [11].

This article aims to review the general trends in PCD-CT research in the cardiovascular field by analyzing articles published over the past five years, focusing on publication trends over the years, different study types published about the topic, country and institution involvement in publications, and the main clinical applications investigated.

## 2. Materials and Methods

### 2.1. Database

The bibliometric analysis data in this study were derived from multiple comprehensive databases, including PubMed, EMBASE, Scopus, and Google Scholar. These databases were selected for their extensive collection of literature relevant to the field of radiology and PCD-CT, ensuring a broad and inclusive dataset for analysis.

### 2.2. Inclusion Criteria and Search Strategy

On the 4th of July 2024, we retrieved all literature related to cardiac photon-counting CT published from 2019 to the present. The search strategies for each database were as follows: For PubMed, the search term used was ((CT angiography) OR (cardiac) OR (CCTA) OR (coronary)) AND (photon-counting detector CT), which yielded 251 records; for EMBASE, the search criteria were (‘CT angiography’/exp OR ‘cardiac’/exp OR ‘coronary computed tomography angiography’/exp) AND (‘photon-counting CT’), which yielded 88 records; for Web of Science, the search term used was TS = (“CT angiography” OR cardiac OR CCTA OR coronary) AND TS = (“photon-counting detector CT”), which resulted in 309 records; and for Google Scholar, when we used the search term “CT angiography” OR cardiac OR CCTA OR coronary AND “photon-counting detector CT”, it resulted in 336 records. After the removal of duplicates, the final number of records included in the analysis was 81. The inclusion criteria for the studies were as follows: involvement of human subjects (clinical), experimental study design (including experimental case series), articles written in English, and use of in vivo datasets. The exclusion criteria included studies involving human phantoms, cadaveric or ex vivo studies, animal studies, reviews, letters, editorials, and case reports.

### 2.3. Data Collection

For each qualified and included record, the following data were collected: country of the first author; year of publication; whether it was a comparison study and, if so, with which imaging modality; the clinical application investigated; list of authors; first author name; institution of the first author; journal name; citation count; number of subjects included in each study (as well as the number of controls); key words; the model of the machine used; and information related to funding.

### 2.4. Data Visualization

This study analyzed various aspects of the collected literature, including the number of articles published annually, the countries and institutions involved, highly cited articles, co-citations, authors, journals, and keywords. The following tools were used for data visualization: Excel 2019 (Microsoft Corp., Redmond, WA, USA) was used to display trends in the number of articles published by year, type of study, journal, country, institution, machine model used, and by clinical application investigated; and VOSviewer version 1.6.20, released on 31 October 2023, was utilized for visualization analysis of the co-occurrence of keywords and authors and to indicate their link strengths [12,13].

## 3. Results

### 3.1. Identification and Selection of Studies

**Figure 1:**
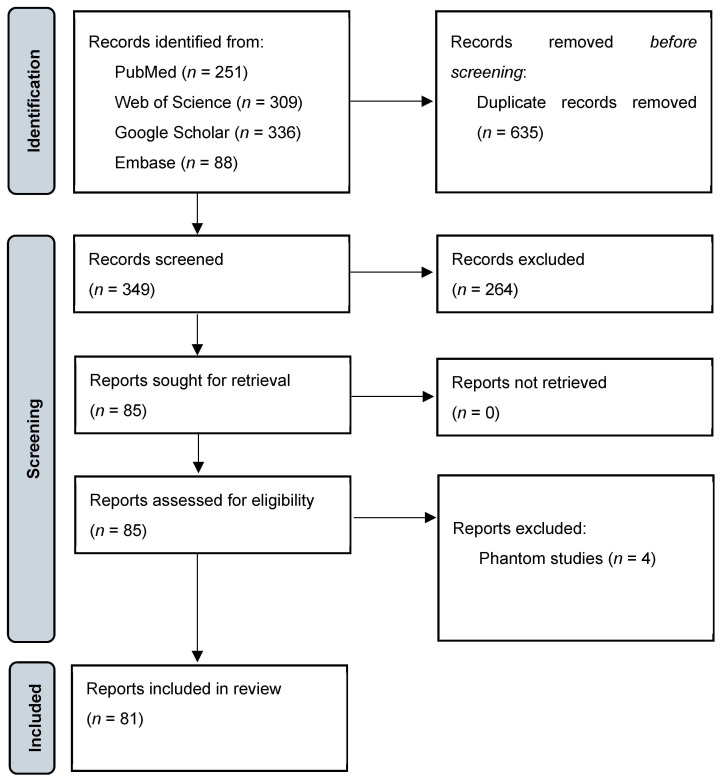
PRISMA flowchart showing the process of study selection [7].

A comprehensive search of multiple databases, including PubMed (*n* = 251), Web of Science (*n* = 309), Google Scholar (*n* = 336), and Embase (*n* = 88), resulted in a total of 984 records. Following the removal of 635 duplicate records, 349 unique records were screened. Of these, 264 records were excluded based on title and abstract review, leaving 85 reports for full-text retrieval. All 85 reports were successfully retrieved and assessed for eligibility, with four being excluded as they were phantom studies. Ultimately, 81 studies were included in the systematic review (see Figure 1) [14].

### 3.2. Trend over Years

**Figure 2:**
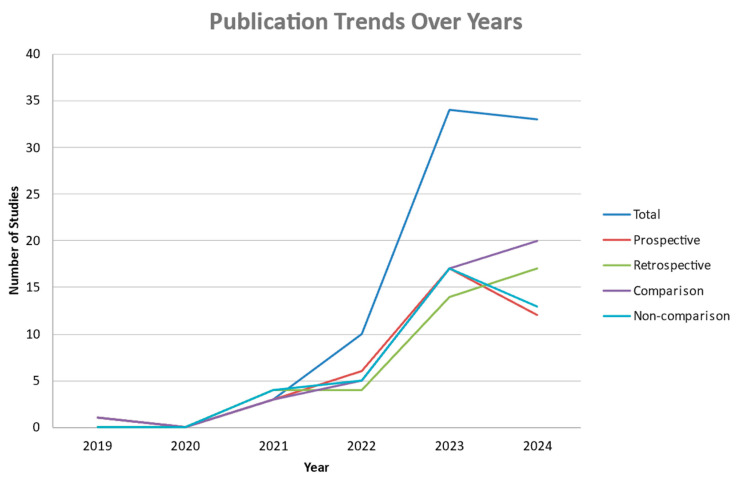
Number of publications in each year from 2019 to 2024 based on the type of study and based on whether it is a comparison study or not, as well as the total publication count depicted in dark blue.

The total number of studies published annually showed an overall clear upward trend within the past 5 years, as illustrated in Figure 2. The publication counts of all different types of studies increased steadily, with a similar pattern peaking in 2023.

### 3.3. Type of Study

**Figure 3:**
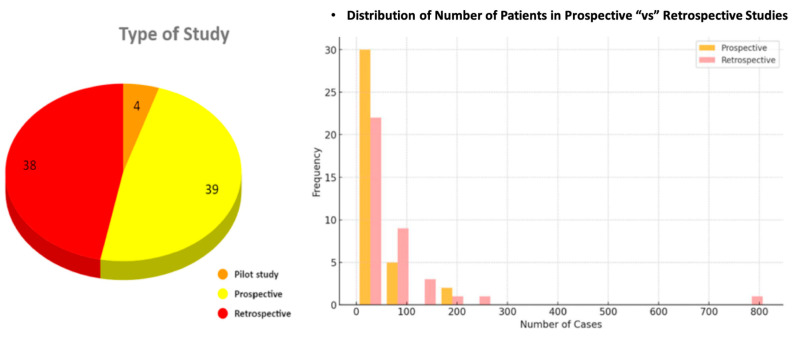
On the left is a pie chart illustrating the number of studies falling under pilot, prospective, and retrospective studies. The diagram on the right illustrates the frequency distribution of the number of cases categorized as retrospective and prospective studies.

The studies were categorized into three types, consisting of 39 prospective studies (48.2%), 38 retrospective studies (46.9%), and 4 pilot studies (4.9%). We also analyzed the distribution of the number of subjects included in the prospective and retrospective studies. This revealed that, in both types of studies, up to 50 patients were included in most studies, with up to 22 retrospective studies and 30 prospective studies falling in this range (see Figure 3).

**Figure 4:**
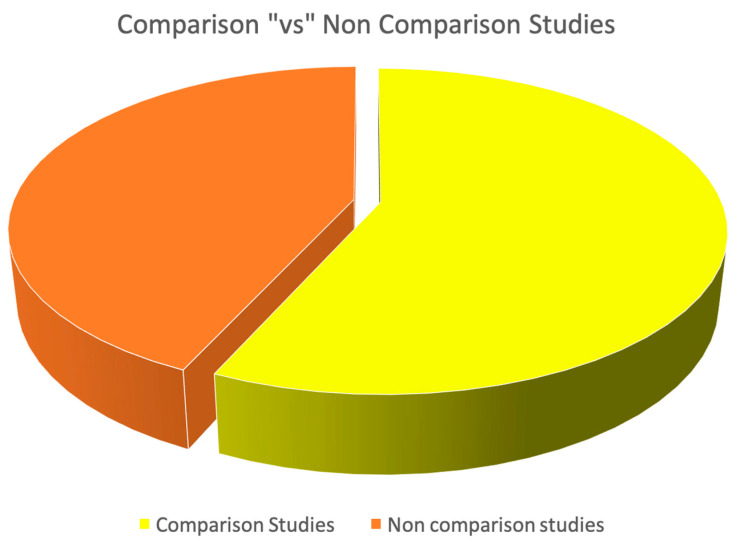
Pie charts depicting the proportion of comparison versus non-comparison studies.

Out of the 81 studies, 46 were comparison studies (56.8%), and 35 were non-comparison studies (43.2%). Additionally, out of the 46 comparison studies, intra-model comparisons accounted for 13 studies (25%), while inter-model comparisons accounted for the remaining 35 studies (Figure 4). The analysis of the collected bibliometric data further showed that most of the inter-modality comparisons were made with standard energy-integrating detector CT, standard high-pitch dual-energy CT, invasive coronary angiography, and MRI.

### 3.4. Country and Institution Involvement

The geographical distribution and institutional involvement along with the number of studies and the total number of citations per country are summarized in Table 1. Institutions from the USA (27 studies), followed by Germany (25 studies) and Switzerland (16 studies) were the most prolific contributors. Notable contributions came from the Mayo Clinic in the USA (10 publications), the University Hospital Augsburg (six publications), and the University of Freiburg in Germany as well as the University of Zurich and Zurich University Hospital in Switzerland (16 publications, 190 citations).

### 3.5. Funding Agencies

Various funding agencies supported the research, with the most frequent contributors being Siemens (18 studies), Projekt DEAL (seven studies), and the European Union (six studies), as detailed in Table 2.

### 3.6. Clinical Applications

**Figure 5:**
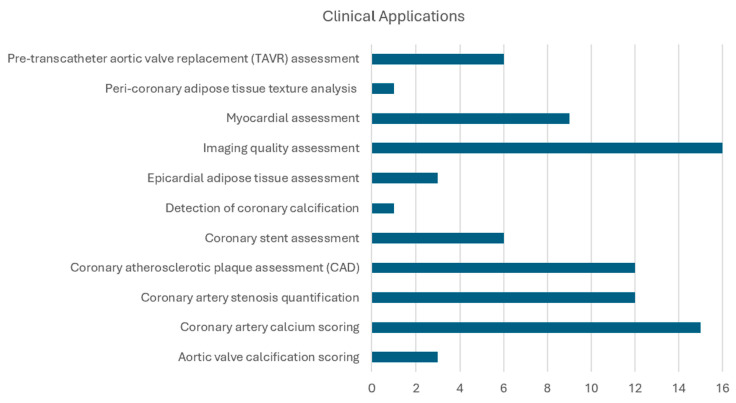
Number of publications for the most common clinical applications discussed in the literature.

The studies addressed a range of clinical applications, with the most frequent applications being image quality assessment (16 studies) and coronary artery calcium scoring (15 studies), followed by coronary atherosclerotic plaque assessment (12 studies) and coronary artery stenosis quantification (12 studies), as shown in Figure 5.

### 3.7. Journals

The included studies were published across a variety of journals, with Radiology (RSNA) contributing ten publications, followed by European Radiology contributing eight publications and Journal of Cardiovascular Computed Tomography and Investigative Radiology contributing seven publications each (see Table 3).

### 3.8. Authors

**Figure 6:**
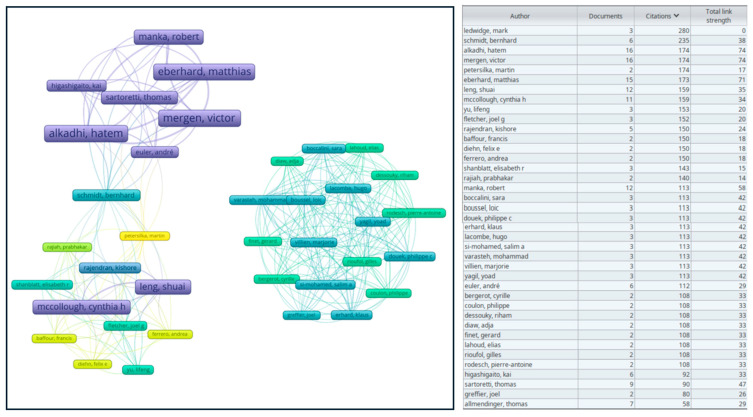
Overlay visualization map of authors on VOSviewer. The size of the boxes is proportionate to the occurrence of each author, and the thickness of the links represents the strength of the association or frequency of cooperation between authors, commonly known as total link strength.

The most productive authors and the most co-cited authors are illustrated in Figure 6. The most productive authors were Mergen V. and Alkadhi H., with 16 publications each, followed by Eberhard M. with 15 publications. Similarly, the total link strength was the highest among the same authors in the same order.

### 3.9. Keywords

**Figure 7:**
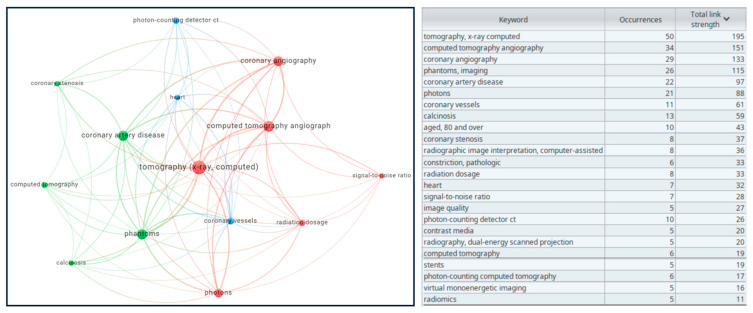
Overlay visualization map of keywords on VOSviewer [5]. The size of the nodes represents the occurrence number of keywords, and the thickness of the links represents the strength of the association or frequency of cooperation between entities, commonly known as total link strength shown also in the table.

A detailed keyword analysis was conducted using VOSviewer, resulting in an overlay visualization that highlights the most common and significant keywords in the included studies (see Figure 7) [7]. This visualization provides insights into the prevalent research themes and emerging trends within the field of CT scan technology.

## 4. Discussion

This bibliometric analysis demonstrates a significant surge in PCD-CT research in cardiac imaging over the past five years. The increasing number of publications, particularly peaking in 2023, underscores the growing interest in PCD-CT and its transformative potential. However, the field remains in its early stages, with significant opportunities for further research and technological improvements. While much attention has been given to the strengths of PCD-CT, it is also important to consider its weaknesses, particularly in relation to applications where traditional EID-CT may still hold advantages.

### 4.1. Global Research Trends and Contributions

Our findings highlight a geographic concentration of PCD-CT research, with leading institutions such as the Mayo Clinic and the University of Zurich playing pivotal roles in advancing the clinical validation and application of this technology.

Countries like the USA, Germany, and Switzerland lead PCD-CT research, benefiting from strong research and development investment (>3% of GDP) and significant healthcare spending (>8000 PPP international dollars per capita) [14,15]. To overcome this imbalance, international collaborations and targeted funding in underrepresented regions are essential. Such efforts could foster a more inclusive research environment and accelerate the clinical translation of PCD-CT worldwide.

### 4.2. Clinical Applications and Impact

Although PCD-CT has been available for clinical use for five years, most studies have primarily focused on its technical performance, including reconstruction methods and image quality. This focus is likely due to the early stage of clinical adoption, during which validating technical robustness takes priority. Additionally, demonstrating clear patient-centered benefits requires long-term, multicenter clinical trials, which are still in progress. As these studies mature, future research should shift toward highlighting the impact of PCD-CT on clinical outcomes and patient care.

Despite the focus of recent literature about PCD-CT on technical issues, the primary clinical applications of PCD-CT, including coronary artery calcium scoring, atherosclerotic plaque assessment, and stenosis quantification, highlight the versatility of PCD-CT in addressing key challenges in cardiac imaging.

Early studies highlight its utility in TAVR planning and follow-up, where its high spatial resolution enhances visualization of the aortic valve leaflets, stents, and vascular access routes. The detailed spectral information provided by PCD-CT enables precise tissue characterization and differentiation, with potential revolution for accurate diagnosis and treatment planning. Spectral reconstruction techniques, such as low-keV virtual monoenergetic imaging and metal artifact reduction, further optimize imaging quality [16,17,18].

**Figure 8:**
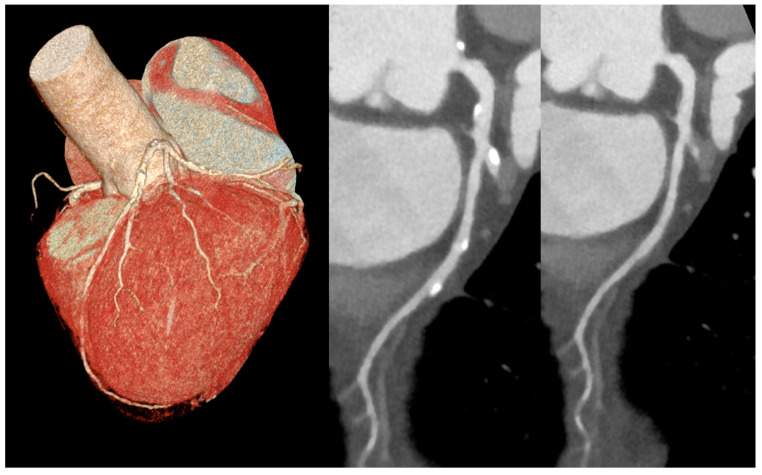
A case of high calcium load over left anterior descending artery in 3D reconstruction (left image). Standard (center image) and pure lumen (right image) reconstruction with spectral virtual removal of calcific lesions, allowing for a more accurate stenosis quantification.

PCD-CT also shows promise for myocardial tissue characterization, with strong correlations for ECV quantification compared to MRI (r = 0.82–0.91, *p* < 0.001), although minor over- or underestimation persists depending on single- or dual-energy modes [19]. Moreover, PCD-CT holds the promise for significant advancements in plaque characterization and stenosis assessment, particularly in patients with dense calcifications or stents, where blooming artifacts are reduced through ultra-high-resolution modes [20,21]. One of the key advancements of PCD-CT is its ability to generate pure lumen images by virtually removing calcific lesions through spectral imaging techniques. This ability enhances the accuracy of stenosis quantification, as illustrated in the case study in Figure 8. The potential of pure lumen imaging is particularly significant for evaluating coronary stenosis, as it provides more precise measurements, reducing the potential for overestimation of stenosis severity due to calcified plaques. The ability to differentiate lipid-rich from fibrotic plaques enhances risk stratification and intervention planning. Additionally, radiomics-based machine learning models applied to PCD-CT angiography have successfully automated the identification of high-risk coronary plaques, demonstrating its advanced diagnostic potential [22].

The ultra-high spatial resolution of photon-counting detector CT angiography allowed for a reclassification of patients toward a lower Coronary Artery Disease Reporting and Data System category compared with standard spatial resolution. Nevertheless, the effects on clinical decision-making, downstream testing, and prognosis must be evaluated in future studies [23,24].

Finally, a critical advancement of PCD-CT is its ability to reduce radiation dose while maintaining high image quality [25]. This is particularly relevant as coronary CT angiography (CCTA) usage rises globally and for monitoring disease progression in longitudinal follow-up studies [26].

However, despite all the discussed strengths of PCD-CT, it is important to acknowledge that some analyses previously possible with EID-CT may no longer be feasible with PCD-CT, particularly in myocardial perfusion imaging. Studies on myocardial perfusion using EID-CT have been instrumental in understanding coronary blood flow dynamics, but no such studies have been conducted with PCD-CT yet. The absence of myocardial perfusion research with PCD-CT remains a significant gap, especially considering that myocardial perfusion was one of the key research trends with EID-CT, and its continued investigation is critical for the full integration of PCD-CT into clinical practice.

Moreover, most of the included studies to date have enrolled fewer than 50 patients; the limited number of patients included in most PCD-CT studies is likely related to the fact that the PCD-CT system was only recently approved for clinical use. As a result, the recruitment of larger cohorts is constrained by the availability of this technology. Future studies should prioritize statistically powered sample size calculations to ensure reliable conclusions and generalizability. This is particularly important for prospective studies, which should carefully adhere to predefined endpoints and include a sufficiently large sample size based on statistical criteria. Future research should prioritize larger, multicenter cohorts exceeding 100 patients to ensure statistically robust conclusions and generalizability.

### 4.3. Limitations

Our study faced several limitations. Firstly, the limited number of studies on clinical PCD-CT imaging constrained our ability to draw definitive conclusions. This challenge is further compounded by the exclusion of articles published later in 2024, though we anticipate continued growth in this research field. The small pool of papers highlights the nascent stage of PCD-CT and underscores the need for more extensive, high-quality research.

Additionally, we did not quantify the funding contributions of individual sponsors, which may have introduced bias. Financial contributions from specific sponsors, particularly those with vested interests, could have influenced the research direction, analysis methods, or interpretation of findings, potentially leading to results that reflect sponsor interests rather than an unbiased evaluation of the data.

Furthermore, our keyword analysis did not account for temporal trends, which could have provided deeper insights into the evolution of research priorities over time. These limitations emphasize the need for larger, more comprehensive studies and richer datasets to fully evaluate the clinical impact and potential of PCD-CT.

## 5. Conclusions

The rapid growth of PCD-CT research underscores its transformative potential in radiological imaging. With superior spatial resolution, enhanced spectral capabilities, and reduced radiation doses, PCD-CT offers significant advancements in diagnostic accuracy and patient outcomes, particularly in cardiac imaging. Currently, the USA leads the field with substantial contributions, but addressing global research imbalances through continued collaboration and targeted investment will be critical. Overcoming existing challenges through larger studies, technological optimization, and international efforts will enable PCD-CT to achieve its full potential and drive further advancements in clinical imaging.

## Figures and Tables

**Table 1:** The geographical distribution and institutional involvement along with the number of studies and the total number of citations per country.

Germany				
	University Medical Center of the Johannes Gutenberg University	4		
	University Hospital Würzburg	1		
	University Medical Centre Mannheim, Heidelberg University	3		
	University Hospital Augsburg	6		
	University of Freiburg	4		
	Other Institutions	7		
	Total Publications	25	Total Citations	161
USA				
	Mayo Clinic	10		
	Medical University of South Carolina	9		
	Duke University	1		
	Other Institutions	7		
	Total Publications	27	Total Citations	405
Switzerland				
	University of Zurich and Zurich University Hospital	16		
	Total Publications	16	Total Citations	190
Hungary				
	Semmelweis University Heart & Vascular Center	7		
	Total Publications	7	Total Citations	10
France				
	University of Lyon	2		
	Other Institutions	6		
	Total Publications	8	Total Citations	220
Netherlands				
	University Medical Centre Rotterdam	4		
	Total Publications	4	Total Citations	15
UK				
	University of Oxford	1		
	Total Publications	1	Total Citations	0
Italy				
	IRCCS Ospedale San Raffaele Milano	1		
	Total Publications	1	Total Citations	0
Belgium				
	University Hospitals Leuven	1		
	Total Publications	1	Total Citations	71
China				
	Shanghai Joao Tong University	1		
	Total Publications	1	Total Citations	0
Japan				
	Okayama University	1		
	Nagoya City University	1		
	Total Publications	2	Total Citations	2
Ireland				
	St Vincent’s University Hospital	1		
	Total Publications	1	Total Citations	0
Sweden				
	Linköping University	2		
	Lund University	1		
	Total Publications	3		0

**Table 2:** Funding agencies that have contributed to funding clinical PCD-CT studies and their respective frequency.

Funding Body	Frequency
Astellas	1
Baden-Württemberg Ministry of Economic Affairs, Labor and Tourism	5
Bard	1
Bayer	5
Berta-Ottenstein-Programme for Clinician Scientists, Faculty of Medicine, University of Freiburg.	1
Bracco	2
British Heart Foundation	2
Elucid Bioimaging	3
European Union	6
G. & J. Bangerter-Rhyner Foundation	2
GE Healthcare	2
German Heart Foundation	3
Guerbet	3
HeartFlow, Inc.	1
Hungarian Academy of Sciences	1
Keya Medical	1
Lund University	1
MAInz-DOC Doctoral College	3
Medical faculty of the University of Augsburg	1
Ministry of Innovation and Technology of Hungary	3
National Research, Development and Innovation Fund (UNKP)	2
National Institutes of Health, USA	5
National Science Foundation of China	1
Onassis Foundation	1
Philips	1
Projekt DEAL	7
Promedica Foundation	1
Siemens	18
Swiss Academy of Medical Sciences	2
Swedish Research Council	1
University of Zurich	2

**Table 3:** The distribution of publications across different journals.

Journal	Number of Publications
Academic Radiology	1
Acta Radiologica	1
AJR	2
BMC Medical Imaging	1
Diagnostic & Interventional Imaging	1
Diagnostics	5
Eur Heart J—Cardiovasc Imaging	1
Eur J Radiol	5
Eur Radiol	8
European Radiol Experimental	2
Frontiers in Cardiovascular Medicine	4
Imaging	1
Int J Cardiovasc Imag	5
International J of Cardiology	1
Investigative Radiol	7
JACC: Cardiovascular Imaging	1
JCCT	7
Journal of Applied Clinical Medical Physics	1
Journal of Clinical Medicine	1
Journal of Computer Assisted Tomography	1
Journal of Thoracic Imaging	1
Medical Physics	1
Nature	1
Physics in Medicine and Biology	1
Proceedings of SPIE, Int Soc Opt Eng	3
Radiology (RSNA)	10
Radiology: Cardiothoracic Imaging	2
The Int J of Med Physics Research & Practice	1

## Data Availability

No new data were created or analyzed in this study. Data sharing is not applicable to this article.
